# Draft genomes of one *Staphylococcus haemolyticus* and five *Staphylococcus lugdunensis* strains isolated from catheterized urine samples of females

**DOI:** 10.1128/mra.00497-24

**Published:** 2024-08-20

**Authors:** Helen Appleberry, Haaris Anjum, Taleah Cage, Kayla Jarm, Haashir Khan, Lizzie Proctor, Junelle Saroca, Alan J. Wolfe, Catherine Putonti, Alex Kula

**Affiliations:** 1 Department of Biology, Loyola University Chicago, Chicago, Illinois, USA; 2 Bioinformatics Program, Loyola University Chicago, Chicago, Illinois, USA; 3 Department of Microbiology and Immunology, Loyola University Chicago, Maywood, Illinois, USA; Department of Biological Sciences, Wellesley College, Wellesley, Massachusetts, USA

**Keywords:** *Staphylococcus haemolyticus*, *Staphylococcus lugdunensis*, urinary microbiome, urobiome, rUTI, OAB

## Abstract

Although *Staphylococcus haemolyticus* and *Staphylococcus lugdunensis* are members of the normal human flora, they also can cause infection. Here, we present the draft genomes of five strains of *S. lugdunensis* and one strain of *S. haemolyticus* isolated from transurethral catheterized urine samples from different females experiencing lower urinary tract symptoms.

## ANNOUNCEMENT

*Staphylococcus haemolyticus* and *Staphylococcus lugdunensis* are coagulase-negative staphylococci (CoNS) and “commensal” members of the skin microbiota (1, 2). However, both are known opportunistic pathogens (3, 4). While *S. haemolyticus* is the second-most frequently isolated CoNS from urine samples, incidences of *S. haemolyticus*-associated urinary tract infections (UTIs) are rising (5). *S. lugdunensis* is recognized as a rare cause of UTIs, while also a part of the nonpathogenic flora of the urobiome (6). Here, we present a *S. haemolyticus* strain, isolated from the urine of a female diagnosed with recurrent UTI (rUTI), and five *S*. *lugdunensis* strains, isolated from five different females with overactive bladder (OAB) symptoms. While prior studies have found instances in which *Staphylococcus* sp. have been more abundant in the urinary microbiota of individuals with OAB, it is unknown if this is associated with the symptoms observed (7, 8).

These urine samples were collected as part of prior IRB-approved studies (see Table 1) (9–13). The strains were cultured from the urine samples by the enhanced quantitative urine culture (EQUC) method (14). Species identification was determined using a matrix-assisted laser desorption ionization-time of flight mass spectrometer (MALDI-TOF MS; Bruker Daltonics, Billerica, MA) as previously described (15), and the isolates were stored at −80°C in the Loyola Urinary Education and Research Collaborative (LUEREC) collection. Samples were retrieved from this collection and streaked on tryptone soy agar (TSA) plates and were incubated in 5% CO_2_ for 24 hours at 35°C. Liquid tryptone soy medium was inoculated with single colonies from these plates and incubated in 5% CO_2_ for 24 hours at 35°C. DNA was extracted from the liquid cultures with the DNeasy Blood and Tissue Kit (Qiagen), following the manufacturer's protocol for Gram-positive species. DNA was sent to SeqCoast (Portsmouth, NH) for library preparation using the DNA Prep tagmentation kit (Illumina) and unique dual indexes. These libraries were then sequenced by SeqCoast on the Illumina NextSeq2000 platform with a 300-cycle flow cell kit (2 × 150 bp reads). The BV-BRC website, v3.35.5 (16), was used for genome assembly with the “auto” parameter. There the reads were trimmed by trim_galore v0.6.5dev (https://github.com/FelixKrueger/TrimGalore), assembled by Unicycler v0.4.8 (17), and polished with Pilon v1.23 (18). Taxonomy was confirmed with the Type Strain Genome Server (TYGS) (19). Assemblies were annotated by the NCBI Prokaryotic Genome Annotation Pipeline (PGAP) v.6.7 (20). Coverage was calculated by BV-BRC; genome completeness and contamination were computed by CheckM v1.2.2 (21) upon submission to NCBI. Default parameters were used unless otherwise specified.

**TABLE 1:** Genome assembly and strain information for the 1 *S. haemolyticus* and 5 *S. lugdunensis* strains isolated from the urobiome

Strain	*S. haemolyticus* UMB3106B	*S. lugdunensis* UMB1735	*S. lugdunensis* UMB5747	*S. lugdunensis* UMB7308	*S. lugdunensis* UMB8915	*S. lugdunensis* UMB8974
No. of Raw Reads	2,584,846	2,130,040	2,186,190	2,157,860	1,915,472	2,971,182
Assembly Length (bp)	2,414,972	2,526,737	2,538,396	2,542,811	2,496,650	2,537,671
G + C (%)	32.74	33.68	33.65	33.64	33.77	33.65
No. of Contigs	62	22	28	27	15	32
Contigs N50 (bp)	106,858	285,955	188,585	369,218	629,127	188,585
Coverage (x)	136.19	119.72	118.19	111.41	103.87	145.19
Completeness (%)	99.31	97.61	97.69	97.66	96.47	97.69
Contamination (%)	0	0.32	0.34	0.34	0.38	0.34
Symptom Status	rUTI	OAB	OAB	OAB	OAB	OAB
IRB Protocol No. (Institution)	170077AW (UCSD)	LU207152 (LUC)	LU207152 (LUC)	LU207152 (LUC)	LU207102 (LUC)	LU207102 (LUC)
Study Reference(s)	11, 12	13	13	13	9, 10	9, 10
SRA Accession No.	SRR28710902	SRR28710913	SRR28710895	SRR28710912	SRR28710910	SRR28710914
Assembly Accession No.	JBCGEY000000000	JBCGEQ000000000	JBCGER000000000	JBCGEP000000000	JBCGEO000000000	JBCGEZ000000000

Information regarding the sequencing of these six strains, *S. haemolyticus* UMB3106B and *S. lugdunensis* UMB1735, UMB5747, UMB7308, UMB8915, and UMB8974, can be found in Table 1. For both species, the number of sequenced isolates from the urinary tract is limited. Further sequencing of *Staphylococcus* isolates from the urine of individuals with OAB is needed to explore associations between symptoms and CoNS species.

## Data Availability

Table 1 lists the SRA accession numbers and assembly accession numbers for the six strains.
